# Insights from the young people who serve on youth advisory councils in Africa: a mixed-methods study

**DOI:** 10.1186/s12889-026-30072-x

**Published:** 2026-10-27

**Authors:** Luwam T. Gebrekristos, Samantha Pagán, Ife Albert, Shakira Choonara, Augustina Mensa-Kwao, David Imbago Jácome, Olivia Biermann, Jason M. Nagata, Luo Li, Molly O’Sullivan, Alex Ezeh, Sarah Baird, Allison K. Groves

**Affiliations:** 1 Department of Medicine Huddinge, Karolinska Institutet, Stockholm, Sweden; 2 Department of Community Health and Prevention, Drexel University, Philadelphia, PA USA; 3 Johannesburg, South Africa; 4 Department of Mental Health, Johns Hopkins Bloomberg School of Public Health, Baltimore, MD USA; 5 Quito, Ecuador; 6 Department of Global Public Health, Karolinska Institutet, Stockholm, Sweden; 7 Department of Pediatrics, University of California, San Francisco, San Francisco, CA USA; 8 Global Adolescent Health, Murdoch Children’s Research Institute, Parkville, VIC Australia; 9 Department of Global Health, Milken Institute School of Public Health, George Washington University, Washington, DC USA

**Keywords:** Adolescents, Youth engagement, Mixed-methods, Young people, Youth participation

## Abstract

**Background:**

Youth Advisory Councils (YAC) are increasingly recognized as key mechanisms to promote meaningful youth engagement. Despite the rapidly growing adolescent population in Africa, existing research on youth engagement largely focuses on barriers to youth engagement in non-African contexts. This mixed-methods study expands understanding of youth engagement by examining who participates in YACs in Africa, how YAC members in Africa define meaningful youth engagement, and what factors facilitate their participation.

**Methods:**

This cross-sectional study draws on quantitative surveys (*n* = 154) and qualitative interviews (*n* = 14) with current or past YAC members living in Africa (aged 18–30 years old). Quantitative data were summarized using descriptive statistics and interviews were analyzed thematically. Findings from both sources were triangulated to identify key patterns.

**Results:**

Participants resided across 15 countries, and the majority were current YAC members (94.2%). Despite diverse experiences within their roles on advisory councils, YAC members shared a common conceptualization of meaningful youth engagement, including co-creating and valuing youth voices. Further, YAC members voiced that co-creating and valuing youth voices is attainable through two key factors: integrating youth perspectives into organizational frameworks and providing key support. However, many participants’ lived experiences as youth advisors fell short of true meaningful youth engagement.

**Conclusions:**

Our findings underscore the importance of youth-led engagement in Africa and organizational support to foster meaningful youth engagement, particularly in light of the continent’s rapidly expanding youth demographic. Engaging and amplifying the voices of adolescents when developing policies and advocating for solutions is essential for both youth development and social and economic outcomes.

**Supplementary Information:**

The online version contains supplementary material available at https://doi.org/10.1186/s12889-026-30072-x.

## Background

Africa is home to a rapidly growing adolescent population, positioning the region as a critical actor in global youth development and policy. By 2050, 36% of all adolescents (aged 15–24 years) will be living in Africa [1, 2], and by the end of the 21st century, nearly half of the world’s adolescents will reside on the continent [3]. Given this growth, engaging young people in Africa is not only vital for their personal development but also for the broader health, socio-economic and political development of adolescents and their communities worldwide.

Youth Advisory Councils (YACs) have emerged as influential mechanisms for fostering meaningful youth engagement within organizations, ranging from multilateral and governmental agencies and non-profit organizations to educational institutions [4–7]. Similarly, youth leadership groups and the inclusion of youth in steering committees are established strategies for organizations to systematically incorporate youth voices in decision-making bodies. YACs can provide platforms for young people to contribute their perspectives and solutions to pressing issues that impact their lives, their communities, and their futures [8]. Collectively, these approaches incorporate youth voices in decision-making bodies and help build and expand the capacity of youth participants [9].

Emerging global research highlights the need for intentional strategies to meaningfully engage youth in co-creation, governance, and management of YACs [10–14]. The second *Lancet *Commission on adolescent health and wellbeing recently highlighted that meaningful youth engagement in research enhances study relevance, quality, and translation into practice, while fostering mutual learning and equity between researchers and young people [15, 16]. Despite growing evidence of impact on youth (e.g., personal development and building social networks) and community (e.g., guiding policy decisions) and increasing calls for meaningful youth engagement, existing literature has identified key barriers to ensuring youth involvement [5, 8, 17–25].

Several frameworks have been developed to conceptualize and advance youth participation in programs and research. Hart’s Ladder of Participation, an influential early framework, placed varying degrees of participation on a ladder, with lower rungs signifying non-participatory forms (e.g., tokenism), and higher rungs representing more desirable forms of participation, including the most desirable: youth-initiated, shared decisions with adults [26]. However, the model’s linear structure has been criticized for implying that greater youth participation is inherently more meaningful and for overlooking the social and environmental contexts in which youth participation occurs [27, 28]. Subsequent frameworks have shifted towards non-hierarchical models of participation that account for context, purpose, and circumstances of youth participation [27]. The Typology of Youth Participation and Empowerment (TYPE) Pyramid describes youth participation as different configurations of youth-adult participation, ranging from youth-led to adult-led [29]. Unlike Hart’s Ladder, the TYPE Pyramid moves away from a strict linear process, while emphasizing that the quality and balance of youth–adult involvement determine the potential for youth empowerment and positive development rather than youth control alone. Finally, building on and extending these prior frameworks, the 7P framework further reframes youth participation as a dynamic process, shaped by seven interconnected domains: purpose, positioning, perspective, power relations, protection, place, and process [30]. Rather than assuming greater youth control leads to empowerment, it emphasizes how context, relationships, safe spaces, and participatory methods shape what participation can achieve. Unlike previous frameworks, the 7P framework recognizes that young people may move between different forms and roles within the same activity as circumstances and power relations shift. Thus, it highlights the fluid and contextual nature of participation and the ways in which power, place, and process shape whose perspectives are heard and how participation unfolds. The Lundy model, a complementary approach to the 7P framework, provides four interrelated criteria: space, voice, audience, and influence, to assess whether youth engagement is meaningful and ensuring that youth voice is given due weight in decision-making [31]. Together, these frameworks suggest that meaningful youth engagement is shaped not only by the degree of youth control, but also by the broader socio-cultural context and intergenerational power relations that structure opportunities for youth voice and influence.

Achieving meaningful youth engagement may be particularly challenging given the age-related power imbalances between adults and youth, which elevate adult voices in decision making [24, 32]. Existing frameworks on youth engagement highlight how these imbalances shape youths’ power in decision-making, with meaningful engagement often requiring additional support and resources. Several studies reveal persistent barriers to youth engagement, including vague scopes of work, tokenism, limited resources, lack of transparency and accountability, negative attitudes from adults, and limited supportive and safe environments [5, 8, 17–24, 29]. Addressing these barriers can open pathways for organizations to successfully facilitate collaboration between adults and youths in their spaces and programs.

Extant literature, while important to understanding YACs, suffers from several limitations when considering their role in Africa. First, the geographic focus is heavily skewed toward non-African contexts, with very limited empirical evidence from African youth. Second, although a few studies examine the concept from the African youth perspective, all studies use small samples and qualitative data [21, 33]. Specifically, a Zimbabwean study explored barriers to youth participation in local governance through focus group discussions with young councilors and two South African studies explored reflections of adolescent advisory research groups and members of a youth policy-making group [21, 33, 34]. The importance of meaningful youth engagement is well-established, and yet, what makes youth engagement meaningful from the youth perspective is underexamined in Africa.

This study aims to address these gaps. Specifically, we expand understanding of meaningful youth engagement in Africa by describing who participates in YACs, exploring what meaningful youth engagement means and understanding what facilitates meaningful youth engagement, drawing on the perspective of YAC members living in Africa.

## Methods

### Study design

This mixed-methods study was designed as part of the second *Lancet* Commission on adolescent health and wellbeing [16] to examine the barriers and facilitators of meaningful youth engagement among YACs in Africa. Youth engagement was a central feature of the second Commission, with ten youth Commissioners (aged 24–35 years old) playing meaningful roles such as leading thematic workstreams and leading the design and implementation of this study.

### Recruitment and study sample

Quantitative surveys and qualitative interviews were employed to explore meaningful youth engagement. Using the professional networks of *Lancet *Youth Commissioners and snowball sampling, we recruited members of YACs to participate in the quantitative survey. For the current study, the analytic sample was restricted to participants residing in Africa (*n* = 154). All survey participants were invited to participate in the qualitative interview, with 92 participants expressing interest in participating in the interview. Of the 92 participants, 18 were randomly selected for interviews, with 14 completing the interviews.

Inclusion criteria for both studies included being a current or past (in the last 5 years) member of a YAC/board/group, youth member of a steering committee or member of a youth leadership group; being 18–30 years old [35], and speaking English. Given that youth engagement strategy and terminology can vary depending on the sector, we included YAC, steering committee and youth leadership group, all of which aim to incorporate youth voices into the decision-making process. Eligibility was not dependent on YAC’s focus (e.g., research, governance). Participants were entered into a raffle to receive compensation for participation at the end of the data collection (with their consent). Ethics approval was obtained from the institutional ethics review board at Drexel University (2301009662). Our findings are reported in line with STROBE guidelines.

### Quantitative data collection

Survey questions were developed by the youth Commissioners, many of whom had previously served as YAC members. Using a 5-point Likert scale, participants were asked how important a set of items were to meaningful youth engagement (Appendix; e.g., the direction and activities of the YAC are completely decided by the YAC members). To understand their experiences in YACs, participants were asked to rate their agreement level for the same items above, also using a 5-point Likert scale. Additional questions were asked to collect sociodemographic information and YAC-related characteristics (e.g., received financial compensation).

### Qualitative data collection

The qualitative interview guide was developed by the youth Commissioners (Appendix). Interviews were conducted via Zoom after quantitative data collection was completed. Interviews ranged from 30 to 90 min in length. Interviews were audiotaped and transcribed. Interviews were conducted by two trained research assistants with prior experience in qualitative data collection. Both interviewers were young women, a Nigerian and an American. The similarity in age to participants may have facilitated rapport and openness. Both interviewers received standardized training to ensure consistency in data collection.

### Analyses

As is typical in concurrent mixed methods studies [36, 37], we applied a triangulation approach to analyze the results. Through this process, we examined and interpreted similarities and differences across the two data sources.

#### Quantitative analysis

We first describe the study participants’ sociodemographic characteristics. Next, we focused on questions about the importance of various elements of meaningful youth engagement. Further, we examined responses related to their YAC experiences.

#### Qualitative analysis

We coded our data in Dedoose software using both deductive and inductive codes. Deductive codes were informed by the interview guide, and inductive codes were informed by emerging concepts from the qualitative data. To enhance the rigor of our coding process, two study team members double coded the first four interviews, which were then discussed extensively in a group meeting (LG, AG, IA, SP), during which the team revised code definitions as needed to ensure consistent application of codes in future rounds. The same two coders then double coded an additional two interviews. Inter-coder reliability was high at the second meeting and thus all remaining interviews were coded by a single author (SP) [37]. Analysis focused on how participants described meaningful youth engagement, the facilitators, and barriers they had experienced, and whether their roles and relationships with their YAC’s larger organization supported meaningful youth engagement. Once we completed coding, we identified patterns in findings across participating youth through matrices, thus ensuring thematic saturation.

## Results

Our sample included 145 youths who were currently involved (Table 1) in a YAC or youth board (94.2%) and 9 youths previously involved (5.8%). The mean age of participants was 25.2 years (range 18–30 years). Approximately half of all participants identified as women (52.0%), and the remainder identified as men (46.1%) or as non-binary (2.0%). Over half of the participants had completed university education (58.4%) and less than one in ten participants had completed postgraduate studies (6.5%). A third of all participants were living in Kenya (*n* = 51; 33.1%). More than one-third of participants were born in an urban setting (42.2%), one-third in a rural setting (37.7%), and one-fifth in a peri-urban setting (20.1%).

**Table 1:** Demographic characteristics of study participants (*n* = 154)

YAC member, %(*n*)	
Past^a^	5.8% (9)
Current	94.2% (145)
Type of YAC member, *%(n)*	
Leader	64.9% (100)
Member	31.8% (49)
Other	2.0% (3)
Missing	1.3% (2)
Age, *mean (SD)*	25.2 (2.7)
Gender, *%(n)*	
Men	46.1% (71)
Women	52.0% (80)
Non-binary	2.0% (3)
Highest level of education,* %(n)*	
Some secondary education	2.6% (4)
Completed secondary education	7.8% (12)
Some university	24.7% (38)
Completed university	58.4% (90)
Completed postgraduate studies	6.5% (10)
Area of birth, *%(n)*	
Urban	42.2% (65)
Peri-urban	20.1% (31)
Rural	37.7% (58)

*YAC* Youth Advisory Council

^a^ Past YAC members were members in the last 5 years

The YACs focused on a variety of topics (Table 2). Approximately half of participants reported that their YAC focused on sexual and reproductive health and rights (59.7%), advocacy (59.1%), health and wellbeing of adolescents and young people (48.7%) or training or capacity building (45.5%). Approximately one-third reported that their YAC had a global focus (31.2%). Two-thirds reported that their YACs were part of a non-profit organization (68.8%).

**Table 2:** Focus of youth advisory councils (YAC)

	% (*N*)
Focus area of YAC	
Sexual and Reproductive Health Rights	59.7% (92)
Advocacy	59.1% (91)
Health and wellbeing of adolescents and young people	48.7% (75)
Training/Capacity	45.5% (70)
Gender equality and women’s rights	39.6% (61)
Mental health	37.0% (57)
Human rights	35.1% (54)
Health and wellbeing of women	29.2% (45)
Health service provision/delivery	28.6% (44)
Climate change and/or environment	21.4% (33)
Government/Policy	17.5% (27)
Peace/Conflict resolution	17.5% (27)
Accountability	16.9% (26)
Health and wellbeing of men	15.6% (24)
Health and wellbeing of children	14.3% (22)
LGBTQ rights	13.6% (21)
Digital health/Artificial intelligence	8.4% (13)
No specific focus	2% (3)
Global focus	
Yes	31.2% (48)
No	68.8% (106)
YAC is part of	
Non-profit organization	68.8% (106)
Multi-lateral organization	13% (20)
Academia	6.5% (10)
Governance group	4.6% (7)
Other	2.6% (4)
Missing	4.6% (7)

*YAC* Youth Advisory Council, *LGBTQ* Lesbian, Gay, Bisexual, Transgender, Queer

^a^ More than 1 choice could be selected

### Conceptualizing meaningful youth engagement

**Fig. 1:**
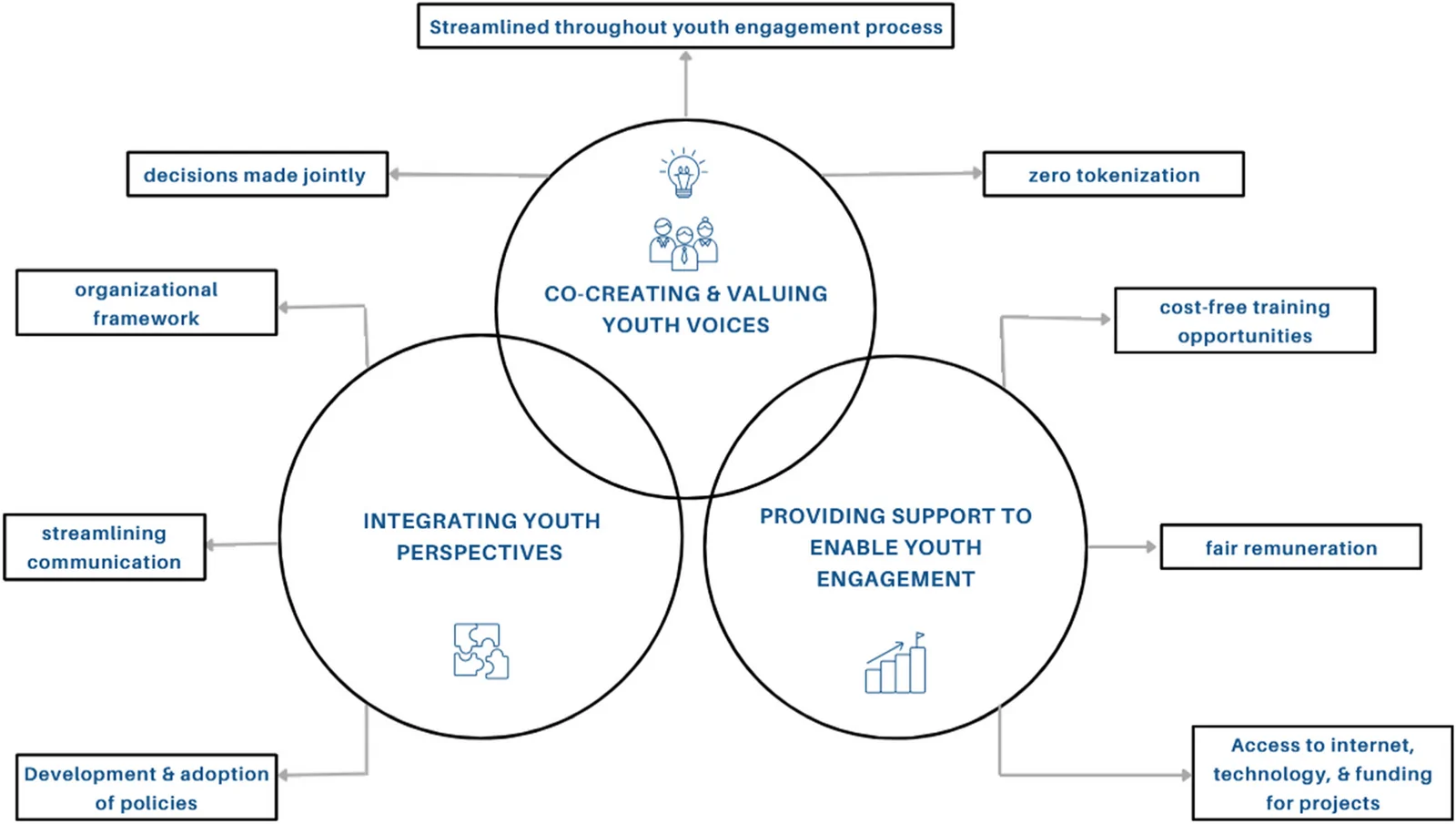
Conceptualizing meaningful youth engagement

We identified one core element, supported by two key factors, that participants felt was critical to meaningful youth engagement (Fig. 1). First, participants described co-creating and valuing youth voices at all stages of an organization’s work process as a key element to meaningful youth engagement. Further, participants described two distinct factors needed to enable co-creating and valuing youth voices. Namely, participants emphasized the need for (1) youth perspectives to be integrated into the broader organizational framework and (2) key support from parent organizations to enable their engagement. In presenting the findings, we describe participants’ definitions of the core element and two key factors while integrating their lived experiences related to each.

### Co-creating and valuing youth voices

According to the qualitative data, a necessary element of meaningful youth engagement is *ongoing co-creation*: including youth at all stages – planning, implementation, and evaluation. Further, youth feel respected when their voices are *valued* without tokenization. That is, youth feel valued when they are represented in decision-making spaces and their contributions are given the same weight and consideration as those of others. A current YAC member (28 years old) from Uganda, YAC Member A, shared their perspective on what a non-extractive engagement experience should look like:

Meaningful youth engagement means…giving youth the opportunity to be at the center of decision-making…You’re able to engage with them, you’re able to listen to them, you’re able to take their…discussions and thoughts and everything into consideration…That’s what I call meaningful engagement, engaging with them at all spheres of your work.

This sentiment is reflected in the quantitative data (Table 3): the majority of respondents believe that their YAC being valued by the larger organization (70.1%) and having decisions on direction and activities made jointly (60.4%) was extremely important to meaningful youth engagement. Conversely, one-third of respondents felt that having decisions made solely by youths was extremely important to meaningful youth engagement (39.6%).

**Table 3:** Core element of meaningful youth engagement: co-creating and valuing youth voices (*N* = 154)

	% (*N*)
YAC is valued by larger organization, %(*n*)	
Extremely important to MYE	70.1% (108)
Sort of important to MYE	12.3% (19)
Neutral	6.5% (10)
Not very important to MYE	0.7% (1)
Not at all important to MYE	0% (0)
* Missing*	10.4% (16)
Direction and activities of the YAC are jointly decided, *%(n)*	
Extremely important to MYE	60.4% (93)
Sort of important to MYE	18.2% (28)
Neutral	9.1% (14)
Not very important to MYE	1.3% (2)
Not at all important to MYE	0% (0)
* Missing*	11% (17)
Direction and activities of the YAC are completely decided by YAC, *%(n)*	
Extremely important to MYE	39.6% (61)
Sort of important to MYE	27.9% (43)
Neutral	18.8% (29)
Not very important to MYE	2% (3)
Not at all important to MYE	0% (0)
* Missing*	11.7% (18)
My involvement in the YAC is valued by the larger organization, *%(n)*	
Strongly Agree	39.6% (61)
Agree	37.7% (58)
Neither agree or disagree	10.4% (16)
Disagree	3.9% (6)
Strongly Disagree	0.7% (1)
* Missing*	7.8% (12)
YAC members are encouraged to speak out at larger meetings, *%(n)*	
Strongly Agree	38.3% (59)
Agree	38.3% (59)
Neither agree or disagree	6.5% (10)
Disagree	6.5% (10)
Strongly Disagree	2.6% (4)
* Missing*	7.8% (12)
YAC agenda meetings are determined by, *%(n)*	
YAC only	52.6% (81)
Larger organization only	4.6% (7)
YAC & larger organization	36.4% (56)
No agenda	0% (0)
* Missing*	6.5% (10)

*YAC *Youth Advisory Council*, MYE *Meaningful Youth Engagement

Youths were asked to reflect on whether their lived experience of serving on a YAC aligned with their definition of meaningful youth engagement, and approximately three-fourths of participants strongly agreed or agreed that their involvement in the youth council is or was valued by the larger organization (77.3%) and/or that they were encouraged to speak out at large meetings (76.6%). Despite nearly two-thirds believing that decisions and activities made jointly is important to meaningful youth engagement, only one-third reported that their meeting agendas are or were co-created by the larger organization and YAC members (36.4%) and the majority of participants reported that agendas were determined by YAC members solely (52.6%).

In the qualitative data, youth expressed feeling tokenized when their YACs were offered a symbolic and/or inconsequential space within an organization. This is reflected by current YAC Member B from Botswana (25 years old) who speaks on the common action of larger organizations to treat youth involvement as a checkbox rather than a meaningful resource:

I’ve come to realize that…young people haven’t been really engaged…in fact, [youth engagement] is a matter of ticking the boxes…I wanted to advocate for young people not to be used as tokens but also to be treated as partners in each and every step of the way of programming and implementation of programs.

### Integrating youth perspectives into an organizational framework

Participants expressed that integrating youth perspectives within an organization’s framework, rather than solely between youth and senior organizational leaders, is key to ensuring co-creation and valuing youth voices. In the qualitative data, participants defined this key factor as their organizations having policies that outline engagement processes and communication between the larger organization and the YAC. Such policies could include consistent, non-sporadic involvement of and communication with the YAC to provide clarity on the ways in which they are to be involved in decision making and advocacy. Current YAC Member C from Nigeria (29 years old) shared their idea of what clear and supportive guidance should look like from an organization:

So, from the get-go, they – whoever is requesting young people’s engagement should be clear on what – what they want from them, right? What is expected of them, it should be totally clear. And then they create that space for them to be able to, you know, deliver on what they want from them. So, if they want this advisory group to be self-run by the young people themselves, like, the young people to sort themselves and to appoint who will be a leader among themself, it should be part of the reference documents, you know, to guide them.

In addition, participants also expressed a desire for ongoing communication from the larger organization regarding YAC’s recommendations. Participants highlighted the importance of implementing youth members’ recommendations to foster meaningful engagement, which reinforces co-creation and valuing youth’s voices (core element of meaningful youth engagement). Specifically, some participants desired policies that establish ongoing communication and implementation of YAC’s recommendations to promote transparency and ensure co-creation between the larger organization and YAC. A current 29-year-old YAC member in Nigeria, YAC Member D, shares that creating something tangible with the information YAC members share with the larger organization is important to the meaningful engagement process:

*Paying attention to what the youth say*,* putting it into account*,* and doing something tangible with the information we’ve shared.*

In the quantitative data, approximately two-thirds of youth reported that the following were extremely important to meaningful youth engagement (Table 4): their voices and expertise are integrated into the larger organization (66.2%), the structure of the organization allowed for streamlined communication (73.4%), and that there was frequent reporting back from the larger organization to the YAC on how their suggestions were being used (64.9%).

**Table 4:** Key factor 1: integrating youth perspectives into an organizational framework (*n* = 154)

	% (*N*)
Voices and expertise of the YAC members are well-integrated into the larger organization’s efforts, %(*n*)	
Extremely important to MYE	66.2% (102)
Sort of important to MYE	14.9% (23)
Neutral	6.5% (10)
Not very important to MYE	0.7% (1)
Not at all important to MYE	0% (0)
* Missing*	11.7% (18)
Structure of the organization allows streamlined communication between the YAC members and the larger organization, *%(n)*	
Extremely important to MYE	73.4% (113)
Sort of important to MYE	9.1% (14)
Neutral	4.6% (7)
Not very important to MYE	1.3% (2)
Not at all important to MYE	0.7% (1)
* Missing*	11% (17)
Frequent report back from larger organization to the YAC members on how their suggestions or input are being used, *%(n)*	
Extremely important to MYE	64.9% (117)
Sort of important to MYE	16.2% (25)
Neutral	6.5% (10)
Not very important to MYE	1.3% (2)
Not at all important to MYE	0% (0)
* Missing*	11% (17)
Voices and expertise of the YAC members are well-integrated into the larger organization’s efforts, *%(n)*	
Strongly Agree	30.5% (47)
Agree	47.4% (73)
Neither agree or disagree	9.1% (14)
Disagree	5.2% (8)
Strongly Disagree	0% (0)
* Missing*	7.8% (12)
Structure of the organization allows streamlined communication between the YAC members and the larger organization, *%(n)*	
Strongly Agree	27.3% (42)
Agree	45.5% (70)
Neither agree or disagree	9.1% (14)
Disagree	9.7% (15)
Strongly Disagree	0% (0)
* Missing*	8.4% (13)
YAC members’ recommendations to the organization are always followed up on, *%(n)*	
Always followed up	35.1% (54)
Very often followed up	22.1% (34)
Sometimes followed up	30.5% (47)
Rarely followed up	5.8% (9)
Never followed up	0% (0)
* Missing*	6.5% (10)
Larger organization takes the contributions of YAC members seriously, *%(n)*	
Strongly Agree	31.2% (48)
Agree	40.3% (62)
Neither agree or disagree	11% (17)
Disagree	9.1% (14)
Strongly Disagree	0.7% (1)
* Missing*	7.8% (12)

*YAC* Youth Advisory Council, *MYE* Meaningful Youth Engagement

When describing their lived experiences, many participants expressed frustration with the larger organization’s failure to implement YAC’s recommendations. They highlighted instances where youth perspectives were sought out but later disregarded. The lack of communication, transparency, and accountability from the larger organization was seen to undermine the youth engagement approach. Member C shared their frustration and recognized the need for an accountability framework to ensure the larger organization kept their YAC updated on how meeting discussions were acted upon:

We [YAC] tell them [larger organization], ‘This is what should be done, or these are the things we recommend,’ like, they don’t get back to the young people to give them feedback on saying…this is what has been done…So – and that was part of the reason why we drafted the accountability framework. But it’s still not – not being, um, utilized or adhered to.

Quantitative findings follow a similar pattern: the majority of respondents strongly agreed or agreed that the voices and expertise of YAC members were well-integrated into the larger organization’s efforts (77.9%), the structure of the organization allows streamlined communication between youths and the larger organization (72.8%), and/or that the larger organization takes the contributions of youths seriously (71.5%). Although three-quarters reported that the structure of the organization allowed for streamlined communication and frequent reporting back between the larger organization and the YAC as extremely important to meaningfully engage with youths, only one-third reported that their YAC’s recommendations were always followed up by the organization (35.1%).

### Enabling youth engagement through support

Participants described support via capacity building, mentoring, and remuneration as another key factor that enables co-creation and valuing youth voices, which fosters meaningful youth engagement. Participants elaborated on the value of these types of support in the qualitative data. Specifically, providing training opportunities and mentorship were critical for youths to feel valued for their contributions and prepared youths to co-create in decision-making spaces. Compensation for time, beyond that of reimbursement for meals and travel, was expressed to be beneficial for morale and the capacity to remain engaged in the YAC. Further, access to and involvement in cost-free training opportunities, group excursions to events such as conferences and/or the larger organization’s offices for skill building, and mentorship from relevant experts were seen as essential ways to create an environment that facilitates youth involvement.

Youth desire mentorship in both personal and professional capacities and seek such support from their larger organizations. Many current YAC participants expected the larger organization to help them develop soft (e.g., time management) and hard skills (e.g., proposal writing) for their future professional endeavors. A 20-year-old past member of YAC in South Africa, Member E, offers insight to how the lack of compensation and inaccessible resources impede a young person’s professional growth:

Young people aren’t compensated, (A) for their efforts and (B) they aren’t even resourced to be in those spaces. So again, meaningful youth engagement is, um, facilitating resources like any other person in the system would have.

Results from the quantitative data were similar to the qualitative findings. That is, the majority of youth said that a larger organization providing some sort of resources to their YAC is extremely important for meaningful youth engagement (Tables 5 and 68.2%).

**Table 5:** Key factor 2: enabling youth engagement through support (*n* = 154)

	% (*N*)
Larger organization provides needed resources and opportunities	
Extremely important to MYE	68.2% (105)
Sort of important to MYE	12.3% (19)
Neutral	7.8% (12)
Not very important to MYE	0.7% (1)
Not at all important to MYE	0% (0)
* Missing*	11% (17)
Larger organization offers opportunities or resources for capacity building for YAC members	
Yes	84.4% (130)
No	8.4% (13)
I do not know	0.7% (1)
* Missing*	6.5% (10)
Received financial compensation	
Yes	36.4% (56)
No	57.1% (88)
* Missing*	6.5% (10)
Thinks compensation is fair^a^	
Yes	57.1% (32)
No	41.1% (23)
* Missing*	1.8% (1)

*MYE* Meaningful Youth Engagement, *YAC* Youth Advisory Council

^a^ Includes only those who received financial compensation

When describing their lived experiences with receiving support and recognition for their work, some participants shared that their larger organization provided resources and mentorship to support YAC activities. A past YAC member from Kenya (29-year-old), Member F, shared their own experience with the liaison from the larger organization their YAC worked with and how that individual supported the YAC members in writing a concept note as a way to help them secure funding for a project they wanted to implement. They explain that while at the time the process was frustrating for the youth, the liaison offered a level of engagement and direct feedback that provided a meaningful learning experience:

So, we’d sit and we’d write and we’d get it wrong and he’d tell us, you know, “This one won’t go through…” And – and so, back then we’d think…he’s becoming a pain…. but, um, even as time went, we realized there was this thing he was teaching us.

In the quantitative data, the majority of participants reported that their organization offers or offered opportunities for capacity building (84.4%). Participants repeatedly voiced the importance of compensation for meaningful engagement in qualitative interviews and quantitative surveys, however, only one-third of participants received financial compensation for their work on the YACs (36.4%). Of those who received financial compensation, approximately half reported that the amount was fair (57.1%).

## Discussion

This mixed methods study expands understanding of meaningful youth engagement by describing the composition of who serves on YACs in Africa as well as defining the concept and key factors from the youth perspective. First, co-creating and valuing youth voices in a non-tokenistic way in all aspects of an organization’s process was identified as a key element of meaningful youth engagement. Second, integrating youth perspectives into an organizational framework is perceived as a key factor for meaningful youth engagement. Specifically, policies that institute streamlined communication and frequent follow ups from the larger organization emerge as key factors in integrating and valuing youth voices and fostering co-creation efforts. Finally, enabling youth engagement by supporting YAC members via capacity building, mentoring and remuneration improves youths’ ability to co-create in decision-making processes. Our findings highlight that integrating youth perspectives into an organizational framework and enabling youth engagement through capacity building, mentoring, and remuneration facilitates the underlying element of meaningful youth engagement: co-creation and valuing youth voices. Despite our findings showing a shared definition for meaningful youth engagement and its key factors, results suggest that experiences of meaningful youth engagement and implementation of key factors were mixed in this population. Specifically, participants described feeling frustrated by the lack of communication and follow-ups from the larger organization. While some participants reported that they received training and mentorship to support their YAC efforts, many reported that they did not receive financial compensation. Our findings align with frameworks, like the 7P framework [29], that conceptualizes meaningful youth engagement not only in terms of the distribution of power and control in decision-making between young people and adults, but also the key factors of youth engagement (e.g., access to resources). Specifically, our findings map onto several interconnected domains of the 7P framework, including purpose and positioning through clear expectations and integration of youth within organizational structures; power relations through valuing youth voices and shared decision-making; and process through co-creation across stages of organizational work. Further, our findings align with the elements of the Lundy model [31], including space and voice by valuing youth voices and enabling youth engagement through capacity building and mentoring; and influence by consistent communication and providing youth with frequent follow ups from the larger organization. However, our findings did not align with some of components of the 7P framework and Lundy model, including perspectives, account for and including diverse experiences and views; place, understanding and adapting to the geopolitical and economic contexts that may influence participation; protection, ongoing attention to the YAC members’ safety and well-being; and audience, ensuring youth are communicating with relevant decision makers. These discrepancies may be because of the population we sampled (e.g., excluding non-English speakers and snowball sampling may have led to a less diverse sample) or the types of questions we explored with participants (e.g., domains around safety and communication patterns with adult decision makers were not explored). Alternatively, it is possible that only some components of prior frameworks are relevant in contexts like the ones were worked in. Future research should examine perspectives, protection, place and audience in greater depth, specifically how the wider socio-cultural context shapes the opportunities for youth to participate and the process to ensure their views are heard and acted on by decision makers in a safe, inclusive and accessible setting. Nevertheless, our study expands knowledge from prior studies (typically examining involvement of African youth in research studies) by showing that access to resources, clear terms of reference, co-designing activities, clear communication, and valuing youth voices throughout the decision-making process, matter not only to those who are in research but are also in non-profit or other settings [20, 33, 34]. Moreover, studies in other regions have identified barriers to meaningful youth engagement, which align with our findings: adults’ reluctance to treating youths as peers, adults’ negative attitudes about youths’ capabilities, and inadequate support [5, 8, 17–23]. Specifically, adults’ reluctance to treat youths as peers and adults’ negative attitudes about youths’ capabilities may indicate a lack of co-creation and valuing youth voices—a key element of meaningful youth engagement according to our findings. Further, adults’ lack of training, negative attitudes about youths’ capabilities, unwillingness to share and concede power, and limited support reflect inadequate capacity building opportunities and mentorship, a key factor of meaningful youth engagement. Our study has several limitations. First, recruitment through professional networks and snowball sampling may have resulted in a non-representative sample of YAC members (e.g., overrepresentation of Kenyan youth), limiting generalizability of results. Second, YAC members were ineligible to participate in the parent study if they were non-English speakers, minors, or did not have internet access. Therefore, our findings may not be generalizable to the broader African YAC population. Further, more than one-half of study participants completed university, which is higher than the average in Africa (i.e., 16% of 18–25 year olds have completed post-secondary education) [38]. The overrepresentation of university-educated individual in our sample may be due to our sampling strategy (i.e., recruiting through professional networks), thereby potentially limiting the generalizability of our findings, or could speak to the non-representative natureof YACs. Findings from a scoping review on YAC in municipal governments indicated that many YAC members were selected by an adult or the larger organization, which may grant preferential treatment and/or create a YAC that is not representative of the community’s youth population [17, 24]. There is growing evidence that YACs may not reflect the community’s youths, thereby excluding marginalized groups (e.g., less educated or rural youth) [17, 21, 24]. As a result, intentional engagement of marginalized youths is needed to ensure their voices are meaningfully and equitably integrated into decision-making. Lasty, while our findings reveal *what* key factors are needed for meaningful youth engagement, *how* to implement the key factors still warrants further research. Our broad sample precludes our ability to explore implementation strategies and processes of YAC. Future studies with YAC and adult decision makers may provide a roadmap to achieving meaningful youth engagement.

Our mixed-methods study broadens our understanding of the core element and key factors of meaningful youth engagement among African YAC members. Consistent with the previous literature, our findings support the importance of co-creation and valuing youth voices (e.g., avoiding tokenization) and support (e.g., capacity building opportunities) in meaningful youth engagement. Our study extends this work by emphasizing the importance of integrating youth perspectives into an organizational framework (e.g., streamlined communication) and remuneration as essential for meaningful youth engagement. Further, our mixed-methods study is a potential exemplar for how to engender meaningful youth engagement. Namely, youth Commissioners led all phases of the study, were integrated into the broader structure of the *Lancet* report, and received mentorship and compensation [16].

## Conclusions

As the youth population grows in the Africa region, it is critical for organizations to recognize youth as key agents of change and prioritize implementing and evaluating their engagement practices with youth to bolster engagement. African youth involved in YACs identified *co-creating and valuing youth voices* as a key element of meaningful youth engagement, which could only be fully attained by *integrating youth perspectives into an organizational framework* and *enabling youth engagement through capacity building*,* support*,* clear communication*,* and remuneration*. Results suggest that experiences of meaningful youth engagement and implementation of key factors were mixed in this population. As the youth demographic continues to increase in this region, it is critical for organizations to implement and evaluate their engagement practices with youth to increase engagement. Meaningful youth engagement is inherently iterative [39], requiring ongoing reassessment and adaptation of roles, frameworks, and support structures as new generations move through YACs. These strategies increase the likelihood that YAC members feel valued as co-creators within organizations whose missions seek to elevate youth-related initiatives.

## Supplementary Information

Supplementary Material 1.

## Data Availability

Data will be made available on request.

## Abbreviations

YAC: Youth advisory council
