# Supplementary material for: Insights from the young people who serve on youth advisory councils in Africa: a mixed-methods study
Source: BMC Public Health. 2026 Oct 27;26:3089. doi: 10.1186/s12889-026-30072-x (PMC13711985; doi:10.1186/s12889-026-30072-x)
Supplement: Supplementary file 1 [file 12889_2026_30072_MOESM1_ESM.docx]

**Appendix**

**Table 1. Items from Quantitative Survey**

| **Importance of Meaningful Youth Engagement** | |
| --- | --- |
| YAC is valued by larger organization | 1= Extremely important to MYE  2= Sort of important to MYE  3= Neutral  4= Not very important to MYE  5= Not at all important to MYE |
| Direction and activities of the YAC are jointly decided |  |
| Direction and activities of the YAC are completed decided by YAC |  |
| Voices and expertise of the YAC members are well-integrated into the larger organization's efforts |  |
| Structure of the organization allows streamlined communication between the YAC members and the larger organization |  |
| Frequent report back from larger organization to the YAC members on how their suggestions or input are being used |  |
| Larger organization provides needed resources and opportunities |  |
| **YAC Experience** | |
| My involvement in the YAC is valued by the larger organization | 1= Strongly Agree  2= Agree  3= Neither agree or disagree  4= Disagree  5= Strongly Disagree |
| YAC members are encouraged to speak out at larger meetings |  |
| Voices and expertise of the YAC members are well-integrated into the larger organization's efforts |  |
| Structure of the organization allows streamlined communication between the YAC members and the larger organization |  |
| Larger organization takes the contributions of YAC members seriously |  |
| **YAC-related Characteristics** | |
| YAC agenda meetings are determined by | 1= YAC only  2= Larger organization only  3= YAC & larger organization  4= No agenda |
| YAC members' recommendations to the organization are always followed up on | 1= Always followed up  2= Very often followed up  3= Sometimes followed up  4= Rarely followed up  5= Never followed up |
| Larger organization offer opportunities or resources for capacity building for YAC members | 1= Yes  2= No  3= I do not know |
| Received financial compensation | 1= Yes  2= No |
| Thinks compensation is fair |  |

**Table 2. Current YAC Member Interview Guide**

| **Topics** | **Questions and Probes** |
| --- | --- |
| Introduction | 1. How many [YAC/Board/Committee/Group] are you currently serving on? 2. How long have you served on each of your [YAC/Board/Committee/Group]? 3. What led you to join your [YAC/Board/Committee/Group]? Probe for past experience in youth engagement, past experience, current work, specifically touching on youth engagement? |
| Understanding the YAC | 4. What is your [YAC/Board/Committee/Group] seeking to accomplish? 5. Are there any adolescent priorities that your [YAC/Board/Committee/Group] focuses on? Which ones? 6. What the geographic focus of your [YAC/Board/Committee/Group]? For example, is it global? Does your work focus on Asian countries? 7. How many total hours a week do you work on your [YAC/Board/Committee/Group]? |
| Understand their involvement with YAC | 8. How can someone join your [YAC/Board/Committee/Group]? Probe for involvement in developing the [YAC/Board/Committee/Group] or ask how the participant was recruited? 9. Tell me about your role within your [YAC/Board/Committee/Group]? Probe for if they hold a leadership role. 10. What achievements have you experienced as a [role] within your [YAC/Board/Committee/Group]? 11. More broadly, what do you or other members think are the achievements of your [YAC/Board/Committee/Group]? 12. What challenges have you experienced as a [role] within your [YAC/Board/Committee/Group]? 13. How did you work through those challenges? Probe for whether they reach out to others. If they did not, why? Probe for coping mechanisms. |
| How the YAC and organization function | 14. Does your [YAC/Board/Committee/Group] runs efficiently? Why?/Why not? Probe for ways the YAC runs/does not run efficiently. 15. What do you think about the way your [YAC/Board/Committee/Group] engages with the organization’s leadership? Probe sufficient level of engagement? How do you engage with your organization? 16. What challenges exist with the [YAC/Board/Committee/Group] and larger organization work together? Probe do you think the YAC and organization work well together? Why or why not? |
| Understand meaningful youth engagement | 17. What does meaningful youth engagement mean to you? 18. Would you consider your YAC as meaningfully engaged in the organization? Why?/ Why Not? 19. What strategies have you found effective in meaningfully engaging youth? 20. {THOSE WHO CONSIDER YAC AS MEANINGFULLY ENGAGED} Can you share examples of meaningful engagement within the [YAC/Board/Committee/Group]? 21. What do you think is the most important thing your organization can do to ensure meaningful engagement with youth? 22. What do you or other [YAC/Board/Committee/Group] members that you work with see as barriers to meaningful youth engagement? 23. How could these barriers be addressed? 24. What do you or other [YAC/Board/Committee/Group] members that you have worked with see as facilitators of meaningful youth engagement? 25. How do you and the youth work in issues of adolescents (e.g., programming, priority setting, advocacy)? |
| Support and capacity building | 26. Can you share any orientation or capacity building programs your organization has for [YAC/Board/Committee/Group] members? Probe for one-on-one mentorship, trainings? Whether these programs provide support for members? 27. What programs, if any, have you participated in?     a. What were the goals of these programs? How often? If they have not participated in programs, why not? 28. Would you recommend these programs to other members? If yes, which ones and why? If no, why not? 29. What did you learn from these programs? How have these programs impacted your involvement in the [YAC/Board/Committee/Group]? 30. What other programs or opportunities would you like to participate in that are not offered? What would you hope to gain from these opportunities? 31. How would these other programs impact your involvement in the [YAC/Board/Committee/Group]? 32. What challenges, if any, did you experience in participating in these programs? 33. What strategies do you or your organization use to support and build capacity among other youth? 34. What are your plans once you leave the [YAC/Board/Committee/Group]? What do you need in order to pursue those plans? In what ways has your YAC provided resources to support your future plans? |
| Composition, Governance | 35. What do you think of the age mix of members in your [YAC/Board/Committee/Group]? 36. What do you think of the gender mix of your [YAC/Board/Committee/Group]? 37. How many members serve on the [YAC/Board/Committee/Group]? 38. What is the governing structure of your [YAC/Board/Committee/Group]? Probe for whether there are elections. Who votes? Number of chairs or leaders? Length of term? How are decisions made? 39. How can new members join? Probe do current members play any role in the process of appointing new members? 40. Does the [YAC/Board/Committee/Group] have a charter, a guiding term of reference? Who drafted the charter? Who can amend the charter? Probe if the YAC had any role in drafting the charter and whether the YAC can amend the charter. 41. How is the [YAC/Board/Committee/Group] accountable to the organization and the youth you represent, i.e. how often do you report; and to who? 42. Are there mechanisms for removing non-performing members of the [YAC/Board/Committee/Group]? Probe if YAC has a role in removing non-performing members. 43. How are priorities set for the [YAC/Board/Committee/Group]? What are the priorities? 44. Are you compensated financially? Do you think the compensation is fair? If not, what would be a fair compensation? |
|  | 45. Do you have anything additional to share? |

**Table 3. Past YAC Member Interview Guide**

| **Topics** | **Questions and Probes** |
| --- | --- |
| Introduction | 1. When were last a part of a [YAC/Board/Committee/Group]? 2. How long did you serve on your [YAC/Board/Committee/Group]? 3. In the past 5 years, did you serve on more than one [YAC/Board/Committee/Group]? 4. What led you to join your first [YAC/Board/Committee/Group]? Probe for past experience in youth engagement, past experience, current work, specifically touching on youth engagement? 5. [if more than 1], What led to you move to another [YACs/Boards/Committee/Groups]? |
| Understanding the YAC | 6. What [was/were] your [YAC/Board/Committee/Group] seeking to accomplish? 7. Were there any adolescent priorities that your [YAC/Board/Committee/Group] focused on? Which ones? 8. What was the geographic focus of your [YAC/Board/Committee/Group]? Probe was it global? Did the work focus on Asian countries? 9. On average, how many hours a week did you worked on your [YAC/Board/Committee/Group]? |
| Understand their involvement with YAC | 8. Tell me about your role within your [YAC/Board/Committee/Group]? Probe for whether they ever held a leadership position. 9. What achievements did you experienced as a [role] within your [YAC/Board/Committee/Group]? 10. More broadly, what did you or other members think were the achievements of your [YAC/Board/Committee/Group]? 11. What challenges did you experienced as a [role] within your [YAC/Board/Committee/Group]? 12. How did you work through those challenges? Probe for whether they reach out to others. If they did not, why? Probe for coping mechanisms |
| How do the YAC and organization function | 13. Did any of your [YAC/Board/Committee/Group] run efficiently? Why?/Why not? Probe for ways the YAC runs/does not run efficiently. 14. What did you think about the way your [YAC/Board/Committee/Group] engages with the organization’s leadership? Probe sufficient level of engagement? How do you engage with your organization’s leadership? 15. How did your [YAC/Board/Committee/Group] and the larger organization work together? Probe positive or negative working relationship? How was it positive/negative? |
| Understand meaningful youth engagement | 16. What did meaningful youth engagement mean to you when you served on [YAC/Board/Committee/Group]? 17. What does meaningful youth engagement mean to you now? 18. What strategies have you found effective in meaningfully engaging with youth? Probe for examples of meaningful engagement within the [YAC/Board/Committee/Group]? 19. What do you see as the most important thing your organization can do to ensure meaningful engagement with youth? 20. What do you see as barriers to meaningful youth engagement? 21. How could these barriers be addressed? 22. What do you see as facilitators of meaningful youth engagement? 23. How did you and the youth work in issues of adolescent (e.g., programming, priority setting, advocacy) while serving on [YAC/Board/Committee/Group]? |
| Support and capacity building | 24. Can you share any orientation or capacity building programs your organization had for [YAC/Board/Committee/Group] members? Probe for one-on-one mentorship, trainings? Whether these programs provided support for members? 25. What programs, if any, did you participate in?     a. What were the goals of these programs? How often? If they have not participated in programs, why not? 26. Did you recommend these programs to other members? If yes, which ones and why? If no, why not? 27. What did you learn from these programs? How did these programs impact your involvement in the [YAC/Board/Committee/Group]? 28. What other programs or opportunities would you have liked to participate in that were not offered? What did you hope to gain from these opportunities? 29. How would these other programs have impacted your involvement in the [YAC/Board/Committee/Group]? 30. What challenges, if any, did you experience in participating in these programs? 31. What strategies did you or your organization use to support and build capacity among other youth? |
| Composition, Governance | 32. What was the governing structure of your [YAC/Board/Committee/Group]? Probe for whether there were elections. Who voted? How were decisions made? 33. Did any of your [YAC/Board/Committee/Group] have a charter, a guiding term of reference? How many did? Who drafted the charter? Who can amend the charter? Probe if YAC had any role in drafting the charter and whether the YAC could amend the charter? 34. How was the [YAC/Board/Committee/Group] accountable to the organization and the youth you represented, i.e., how often did you report; and to who? 35. Were there mechanisms for removing non-performing members of the [YAC/Board/Committee/Group]? Probe if YAC had any role in removing non-performing members 36. How were priorities set for the [YAC/Board/Committee/Group]? What are the priorities? 37. Were you compensated financially? Probe if it was in all [YAC/Board/Committee/Group] they served on. If compensated throughout tenure? Did compensation feel fair? If not, what would be fair? |
| Transition | 38. Why did you leave your [YAC/Board/Committee/Group]? *Ask for each one they served on.* 39. What were your plans once you left the [YAC/Board/Committee/Group]? What did you need in order to pursue those plans? In what ways had your YAC provided resources to support your future plans? |
|  | 40. Do you have anything additional to share? |
